# PTEN Depletion Increases Radiosensitivity in Response to Ataxia Telangiectasia-Related-3 (ATR) Inhibition in Non-Small Cell Lung Cancer (NSCLC)

**DOI:** 10.3390/ijms25147817

**Published:** 2024-07-17

**Authors:** Victoria L. Dunne, Mihaela Ghita-Pettigrew, Kelly M. Redmond, Donna M. Small, Sinéad Weldon, Clifford C. Taggart, Kevin M. Prise, Gerard G. Hanna, Karl T. Butterworth

**Affiliations:** 1 Patrick G Johnston Centre for Cancer Research, Queen’s University Belfast, Belfast BT9 7AE, UK; m.ghita@qub.ac.uk (M.G.-P.); kelly.redmond@qub.ac.uk (K.M.R.); d.small@qub.ac.uk (D.M.S.); k.prise@qub.ac.uk (K.M.P.); k.butterworth@qub.ac.uk (K.T.B.); 2 Airway Innate Immunity Research Group (AiiR), Wellcome Wolfson Institute for Experimental Medicine, Queen’s University Belfast, Belfast BT9 7AE, UK; s.weldon@qub.ac.uk (S.W.); c.taggart@qub.ac.uk (C.C.T.); 3 Northern Ireland Cancer Centre, Belfast Health and Social Care Trust, Belfast BT9 7AB, UK; g.hanna@qub.ac.uk

**Keywords:** non-small cell lung cancer, PTEN, radiotherapy, ATR inhibition, DNA damage, radiation pneumonitis

## Abstract

Radiotherapy (RT) treatment is an important strategy for the management of non-small cell lung cancer (NSCLC). Local recurrence amongst patients with late-stage NSCLC remains a challenge. The loss of PTEN has been associated with radio-resistance. This study aimed to examine the efficacy of RT combined with ataxia telangiectasia-mutated Rad3-related (ATR) inhibition using Ceralasertib in phosphatase and tensin homolog (PTEN)-depleted NSCLC cells and to assess early inflammatory responses indicative of radiation pneumonitis (RP) after combined-modality treatment. Small hairpin RNA (shRNA) transfections were used to generate H460 and A549 PTEN-depleted models. Ceralasertib was evaluated as a single agent and in combination with RT in vitro and in vivo. Histological staining was used to assess immune cell infiltration in pneumonitis-prone C3H/NeJ mice. Here, we report that the inhibition of ATR in combination with RT caused a significant reduction in PTEN-depleted NSCLC cells, with delayed DNA repair and reduced cell viability, as shown by an increase in cells in Sub G1. Combination treatment in vivo significantly inhibited H460 PTEN-depleted tumour growth in comparison to H460 non-targeting PTEN-expressing (NT) cell-line-derived xenografts (CDXs). Additionally, there was no significant increase in infiltrating macrophages or neutrophils except at 4 weeks, whereby combination treatment significantly increased macrophage levels relative to RT alone. Overall, our study demonstrates that ceralasertib and RT combined preferentially sensitises PTEN-depleted NSCLC models in vitro and in vivo, with no impact on early inflammatory response indicative of RP. These findings provide a rationale for evaluating ATR inhibition in combination with RT in NSCLC patients with PTEN mutations.

## 1. Introduction

Lung cancer remains a major global health burden that has the highest incidence rate (11.6% of new cases) and is the most frequent cause of cancer-related deaths (18.4%) [1]. Approximately 85% of lung cancer cases are non-small cell lung cancer (NSCLC), with the majority of patients diagnosed at an advanced stage of the disease [2,3]. Despite major advances in novel therapies and RT, the overall survival for patients has improved only modestly and remains limited by the emergence of therapeutic resistance and disease progression [4,5,6,7]. Consequently, there is a need for the development of novel therapies with improved efficacy in defined groups of patients harbouring specific mutations [8].

Phosphatase and tensin homolog (PTEN) is a tumour suppressor that serves as a negative regulator of the PI3K signalling pathway [9]. In NSCLC, whilst the frequency of PTEN mutations is low (approximately 2–7%), loss of the PTEN protein has been reported in approximately 40% of cases [10,11,12]. Furthermore, the downregulation of PTEN has been associated with reduced progression-free and overall survival, along with increased resistance to standard therapies, such as chemotherapy and radiotherapy [13,14,15]. More recently, PTEN has also been reported to play an important role in the DNA damage response by regulating RAD51 expression, which is essential for double-strand break (DSB) repair [16,17].

RT alone or in combination with chemotherapy is a major treatment strategy in the management of patients with locally advanced lung cancer that are unsuitable for surgery [18]. Cancer cells respond to radiation-induced DNA damage by triggering the DNA damage response signalling pathway (DDR), which plays an integral role in maintaining genomic stability by activating cell cycle checkpoints and initiating DNA damage repair to allow cell survival [6]. Ataxia telangiectasia-mutated (ATM) and Rad3-related (ATR) belong to the class-IV phosphatidylinositol 3-kinase (PI3K)-related family of serine/threonine kinases and are critical components of the DDR [19]. The activation of either of these kinases is dependent on the type of DNA lesion, as ATM responds primarily to DSBs, and ATR regulates intrinsic replication stress [20,21].

Tumour radioresistance has been associated with DDR pathway upregulation, which may result in treatment failure [22]. The selective targeting of components of the DDR, including ATM and ATR, are attractive targets for small molecule inhibition in order to overcome radioresistance and enhance the efficacy of RT [23,24]. This concept has been explored in pre-clinical studies that have targeted ATM and ATR and have proven to synergise with RT [25,26,27]. Furthermore, this potential treatment strategy has translated to a phase 1 platform study in the CONCORDE trial, which aims to assess the safety of multiple inhibitors of the DDR in combination with conventional RT in locally advanced NSCLC patients unfit for concurrent chemo-RT [28].

Synthetically lethal interactions have been well established for Poly(ADP-ribose) polymerase (PARP) inhibitors in tumours with BRCA1/2-mutations in homologous recombination (HR) [29]. Several PARP inhibitors have been approved for use in BRCA1/2 mutated breast and ovarian cancer [30,31,32]. Recent studies have demonstrated a synthetically lethal interaction of PTEN loss with the inhibition of ATM or ATR in prostate and breast cancer cell lines [33,34,35]. Based on these data, alongside the established role of PTEN in the DDR, we hypothesised that ATR inhibition could overcome radiation resistance in PTEN-depleted NSCLC models.

In the current study, we show that ceralasertib enhances RT response in PTEN-depleted cells in vitro, resulting in delayed DSB repair. The combination of ceralasertib with RT was well tolerated in mice, delaying tumour growth compared to each monotherapy. These data support the combination of ceralasertib with RT as a synthetically lethal approach in PTEN-depleted NSCLC.

## 2. Results

The depletion of PTEN was validated in H460 and A549 isogenic cell models using Western blotting. The loss of PTEN resulted in oncogenic activation of S345-phosphorylated CHK1 in both H460 and A549 PTEN-depleted cells, which was not observed in NT cells (Figure S3).

The IC_50_ value of ceralasertib for H460 and A549 isogenic PTEN-depleted cell models was examined using clonogenic survival assays (Figure S1 and Table S1). For all cell lines, ceralasertib reduced clonogenic survival in a dose-dependent manner, however, PTEN-depleted NSCLC cell lines had the greatest reduction in clonogenic survival in comparison to NT counterparts (0.12 µM and 0.16 µM vs. 0.19 µM and 0.19 µM for H460 and A549 PTEN-depleted vs. H460 and A549 NT cells, respectively). For in vitro combination studies, a concentration of 100 nM was chosen for all cell lines.

**Figure 1:**
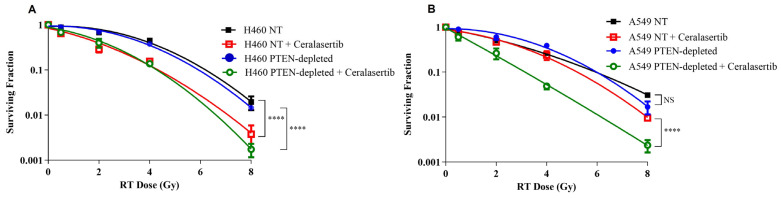
Clonogenic survival curves of (**A**) H460 and (**B**) A549 PTEN isogenic cell models following radiation (■, ●) or combined with 100 nM of ceralasertib in combination with a radiation dose of 0.5, 2, 4, or 8 Gy (□, ○). Each value represents the mean from three independent experiments and the respective standard error. The *p* value for one-way ANOVA was **** *p* ˂ 0.0001 for H460 and A549 cell lines. Statistical differences between groups were calculated using the Student’s *t*-test.

Next, we investigated the impact of ceralasertib on radiosensitivity in NT- and PTEN-depleted cells (Figure 1A,B). The radiobiological parameters for each of the curves in Figure 1 are summarised in Table S2. DEFs were calculated at 10% survival and showed ceralasertib-enhanced radiosensitivity in H460 and A549 NT cells with DEFs of 1.5 and 1.03, respectively (Table S2). Similarly, PTEN-depleted cells showed high levels of radiosensitization with DEFs of 1.32 and 1.73 in H460 and A549 cells, respectively.

The RER was also used to determine radiosensitisation (Table S3). For H460 and A549 cell lines, the combination of ceralasertib with RT significantly enhanced radiosensitivity in PTEN-depleted NSCLC cells in comparison to NT cells (RER = 2.17 (*p* ˂ 0.01); RER = 4.05 (*p* ˂ 0.01), respectively).

**Figure 2:**
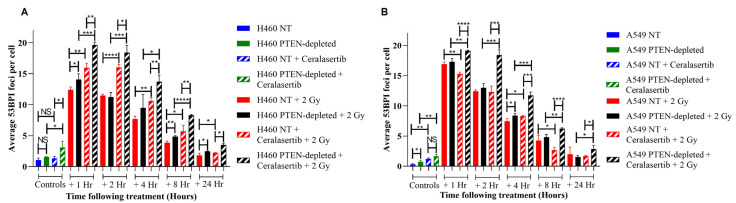
DNA damage repair plotted as mean 53BP1 foci per cell in (**A**) H460 and (**B**) A549 isogenic cell models basally and following 2 Gy alone or in combination with 100 nM of ceralasertib at 1, 2, 4, 8, and 24 h after treatment. Each value represents the mean from three independent experiments and the respective standard error. Differences between two groups were compared by using an unpaired Student’s *t*-test (**** *p* < 0.0001, *** *p* < 0.001, ** *p* < 0.01, and * *p* < 0.05, non-significant (NS)).

To investigate if enhanced radiosensitivity and DNA DSB damage repair are PTEN-dependent, 53BP1 foci were quantified in PTEN isogenic models (Figure 2). Following treatment with ceralasertib alone, 53BP1 foci were elevated in PTEN-depleted H460 cells in comparison to H460 NT cells (*p* ≤ 0.05). When irradiated at 2 Gy, both cell models exhibited a peak in 53BP1 foci at 1 h, which decreased in a time-dependent manner over 24 h. At 1 h, 8 h, and 24 h after irradiation, H460 PTEN-depleted cells had significantly higher mean numbers of 53BP1 foci compared to NT cells (*p* ≤ 0.05). However, no significant difference was observed at 2 h or 4 h post-irradiation. Similarly, A549 PTEN-depleted cells had a greater number of 53BP1 foci 4 h after irradiation (*p* ≤ 0.05) (Figure 2A,B).

Compared to RT alone, combined treatment significantly increased 53BP1 foci at all time points in H460 NT cells (*p* ≤ 0.05). In comparison, a significant increase in 53BP1 was only observed at 4 h in A549 NT cells (*p* ≤ 0.05). Similarly, the loss of PTEN in both H460 and A549 cells resulted in significantly higher mean levels of 53BP1 foci after ceralasertib and RT treatment compared to RT alone across all time points (*p* ≤ 0.05). Furthermore, combined treatment significantly increased 53BP1 foci in H460 and A549 PTEN-depleted cells relative to NT cells at all time points (*p* ≤ 0.05) (Figure 2A,B).

**Figure 3:**
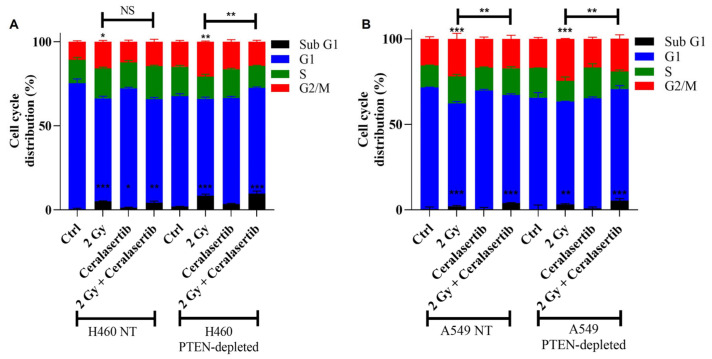
Cell-cycle distribution of (**A**) H460 and (**B**) A549 PTEN isogenic cell models using Propidium iodide (PI) flow cytometry. Cells were treated with ceralasertib and 2 Gy irradiation, alone and in combination and harvested at 48 h. Bars represent the percentage cell-cycle distribution mean of three independent experiments and the respective standard error. Differences between two groups were compared by using an unpaired Student’s *t*-test (*** *p* < 0.001, ** *p* < 0.01, * *p* < 0.05, non-significant (NS)).

Next, we examined the impact of PTEN status on the cell cycle distribution after treatment with ceralasertib alone and combined with RT (Figure 3). RT alone led to an accumulation of cells in sub G1 irrespective of PTEN status. Across all cell lines, ceralasertib alone significantly increased the sub G1 cell population in only H460 NT cells (*p* ≤ 0.05). Combined treatment with RT and ceralasertib significantly increased the proportion of sub G1 cells, with the greatest increase observed in PTEN-depleted H460 and A549 cells compared to NT controls (9% vs. 4%, *p* ≤ 0.01; 5% vs. 3%, *p* ≤ 0.05, respectively).

Similarly, RT caused an accumulation of cells in G2/M in H460 NT- and PTEN-depleted models in comparison to the controls (10% vs. 15%, *p* ≤ 0.05; 15% vs. 20%, *p* ≤ 0.01, respectively). A similar trend was observed in A549 cells with and without PTEN, whereby RT significantly increased the G2/M population (15% vs. 22%, *p* ≤ 0.001; 17% vs. 24%, *p* ≤ 0.001, respectively). However, combination treatment significantly reduced radiation-induced G2/M accumulation. The greatest absolute reduction in radiation-induced G2/M accumulation by ceralasertib was observed in H460 cells with a loss of PTEN in comparison to NT cells (*p* = 9.6 × 10^−5^ vs. *p* = 0.07).

**Figure 4:**
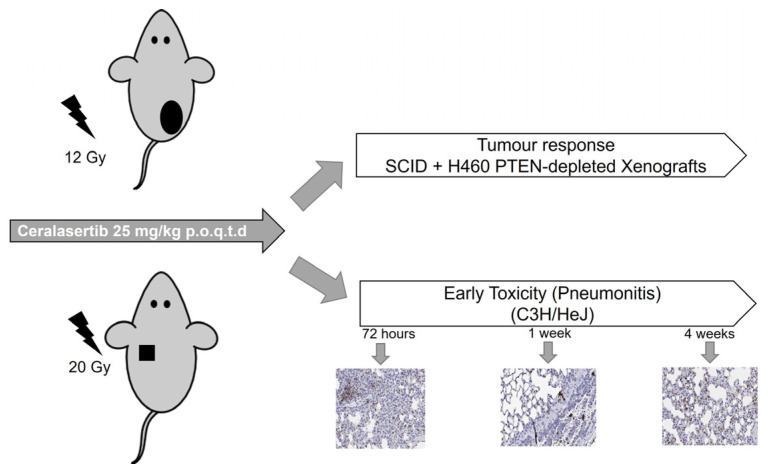
Schematic overview of the preclinical study design. All mice received ceralasertib (25 mg/kg delivered by oral gavage once per day, p.o.q.d) prior to radiotherapy. Tumour response studies were initiated in mice bearing CDXs at a volume of 100 mm^3^ and irradiated with 12 Gy delivered as a single fraction. RP was evaluated after mice received 20 Gy in the upper left lung. Macrophages and neutrophils were assessed at 72 h, 1 week, and 4 weeks post-treatment. 10× magnification was used for representative images.

**Figure 5:**
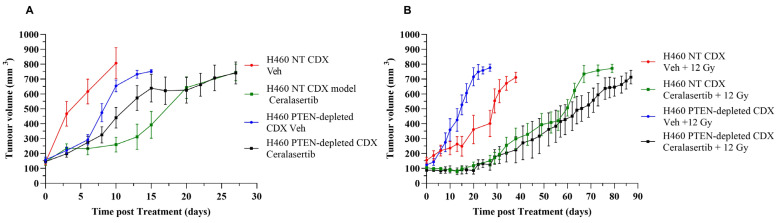
Relative tumour volume curves for H460 NT- and PTEN-depleted CDXs. (**A**) Mice were treated with either drug vehicle or ceralasertib (25 mg/kg per day for 28 days). (**B**) Mice received radiotherapy and radiotherapy in combination with ceralasertib. Irradiation of tumours was performed as a single dose fraction of 12 Gy. Data are presented as median tumour volume for the experimental group ± standard error.

We then investigated the efficacy of ceralasertib and RT on PTEN deficiency using an H460 PTEN-depleted xenograft model. A schematic overview of the study design is described in Figure 4 and is an extension of previous work carried out [26]. Animals were treated with vehicle or ceralasertib alone (28 days × 25 mg/kg) or in combination with 12 Gy based on data from a previous study [26]. The effect of these treatment regimens on tumour growth delay parameters for each of the experimental groups is summarised in Table 1 and plotted in Figure 5.

As monotherapies, ceralasertib and RT enhanced the tumour quadrupling time in both NT (16 days; 22 days, respectively) and PTEN-depleted (2 days; 5 days, respectively) CDX models in comparison to vehicle-treated mice. A significantly greater tumour growth delay was observed in NT CDX xenografts for both treatment regimens (*p* ≤ 0.001). However, the greatest tumour growth delay was observed after a combination treatment of ceralasertib and RT was administered, with PTEN-depleted tumours having a greater quadrupling time in comparison to PTEN-expressing tumours (70 days; 52 days, respectively (*p* ≤ 0.0001)).

**Figure 6:**
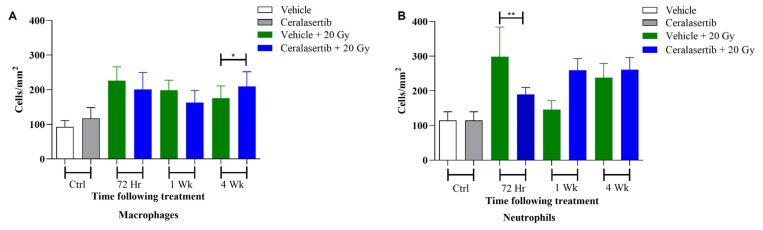
Quantitative analysis of macrophages and neutrophils, early inflammatory response markers indicative of RP. Stained lungs were electronically scanned, and five images were scored from the upper region of the left lobe. Inflammation was defined by the presence of (**A**) macrophage and (**B**) neutrophil cell infiltration, and sections were scored for the number of inflammatory cells present in a 0.25 mm^2^ area at 10× magnification by independent and blind observers. Vehicle-treated mice were compared to ceralasertib alone and in combination with 20 Gy single fraction irradiation at time intervals up to 4 weeks post-treatment. Data are presented as the pneumonitis score ± SEM. Differences between two groups were compared by using an unpaired Student’s *t*-test (** *p* < 0.01, * *p* < 0.05).

To assess the impact of ceralasertib combined with RT on the early inflammatory response in normal lung tissue, we analysed the levels of F4/80 macrophages and NIMP-R14 neutrophils in the irradiated area of the upper left lung (Figure 4 and Figure 6). As a single agent, ceralasertib caused no significant increase in immune cell counts in an area of 0.025 mm^2^ compared to drug vehicle-treated animals. After exposure to a single dose of 20 Gy or in combination with ceralasertib, all animals had an increased number of neutrophils and macrophages per unit area. RT, in combination with ceralasertib, caused a significant increase in macrophage infiltration 4 weeks post-treatment (*p* ≤ 0.05) (Figure 6A) and a significant decrease in neutrophil infiltration 72 h post-treatment (*p* ≤ 0.01) (Figure 6B). However, no other statistically significant differences in macrophage or neutrophil cell infiltration were determined at the other time points when vehicle and RT versus ceralasertib and RT were compared. Representative images of macrophage and neutrophil staining are presented in Figure S2.

## 3. Discussion

There is an urgent need to develop effective therapeutics to improve the prognosis and survival of patients with NSCLC. Previously, we have shown that ATR inhibition using ceralasertib improves the radiation response in preclinical models of NSCLC by enhancing tumour efficacy with no significant impact on radiation fibrosis [26]. In this study, we aimed to develop this treatment paradigm in models representing patients with a loss of PTEN, a multifunctional tumour suppressor gene that is commonly downregulated in NSCLC and associated with radioresistance and, ultimately, poor clinical outcomes [14].

PTEN status has been reported as a prognostic marker in cancer, as its loss can result in resistance to standard-of-care therapies, including chemotherapy and RT [36]. In the present study, clonogenic survival data (Figure 1) and in vivo tumour response data (Figure 5) identified no significant increase in radioresistance in PTEN-depleted NSCLC models. Similar to our findings, an earlier study determined that the PTEN status did not alter the response to RT [37]. Several other studies have reported that a loss of PTEN increases radiosensitivity [38,39], whilst other pre-clinical studies have reported radioresistance in cell lines with PTEN absence [40,41]. Consequently, this suggests that the role of PTEN in the radiation response in cell models is complex and context-dependent.

Despite PTEN status having little impact on radio response, we determined that targeting the DDR under therapeutic conditions influences the radiation response in the context of PTEN status. In this study, ceralasertib induces synthetic lethality and increases the radio-sensitisation in PTEN-depleted NSCLC cells with a cooperative antitumour effect in vivo (Figure 5). In agreement with our findings, other studies have exploited DNA-repair-targeted therapies to be selectively synergistic in specific cancer types with PTEN loss, indicating that PTEN loss may represent an actionable vulnerability for the development of more effective treatment strategies [33,34,35]. This has been attributed to reduced DNA DSB repair due to defects in HR which in turn sensitises PTEN-deficient cancers to DDR-targeted therapies [33,35]. Our results support these early studies as we observed a delayed repair of 53BP1 foci after combined radiation exposure and ATR inhibition in PTEN-depleted cells in comparison to NT cells (Figure 2). We speculate that the accumulation of DNA lesions activates death signals, which, in turn, reduce cell survival. This is supported by our observations that following PTEN loss, ceralasertib and RT combined resulted in an accumulation of cells in Sub G1 (Figure 3).

Despite DDR inhibitors exhibiting promising radio-sensitising effects in tumours, their effect on normal tissue toxicity remains to be elucidated clinically. Previously, we have shown that combining ceralasertib with X-ray treatment has no significant impact on radiation-induced fibrosis in C57BL/6 mice, as measured by histological scoring (*p* > 0.5) and changes in lung tissue density by CBCT (*p* > 0.5) at 4, 12, 26, and 30 weeks [26,42]. Macrophages and neutrophils are the predominant early immunological biomarkers indicative of RP in the lungs. Consistent with our results, several earlier studies have shown similar increases in macrophages and neutrophils in response to radiation exposure [43,44,45]. The current study identified significant changes in macrophages and neutrophils at certain time points after combination treatment in comparison to RT alone. These findings indicate that ceralasertib plus RT may potentiate an early inflammatory response in the lungs; however, it has been previously reported that reducing ATR expression by 10% has a minimal impact on normal tissue homeostasis in mice [46]. Despite this, ATR inhibition has been shown to have strong immunomodulatory effects within the radiation-induced microenvironment; therefore, further studies are essential to fully characterise the interaction between ATR inhibition and RT on acute pneumonitis.

To address concerns surrounding tissue toxicity in patients, the CONCORDE clinical trial will investigate combinations of RT with systemic therapy for NSCLC [28,40]. This multi-arm Phase IB platform study will assess the safety profile of five DDR inhibitors in combination with RT in NSCLC patients with stage IIB/III stages of the disease. Furthermore, this clinical trial will establish recommended Phase II doses (RP2Ds) and assess toxicities that may arise as a result of combination treatment [28,47].

## 4. Materials and Methods

### 4.1. Cell Lines, Drugs, and Reagents

H460 and A549 cell lines were purchased from the American Tissue Culture Collection (ATCC, Manassas, VA, USA). The PTEN-depleted H460 and A549 models were generated using HuSH-small hairpin (shRNA) PTEN constructs in pGFP-V-RS vector (Origene, Rockville, MD, USA) as described by Maxwell et al., 2012 [48]. Briefly, H460 and A549 cells (1.5 × 10^6^) were incubated in a transfection mix that contained plasmid (4 μg), Opti-MEM^®^ medium (Life Technologies, Carlsbad, CA, USA) and Lipofectamine 2000. After 24 h, the medium containing the transfection reagents was removed and replaced with either RPMI-1640 or DMEM medium containing 1 mg/mL of puromycin (ThermoFisher Scientific, Waltham, MA, USA). Individual colonies were selected and expanded, followed by PTEN expression being validated by Western blotting. For all experiments, cells were maintained in their respective mediums, which contained 1 mg/mL of puromycin. Cell culture conditions are described in the Supplementary Material.

Ceralasertib was provided free of charge by AstraZeneca (Cambridge, UK). AstraZeneca had no influence in the concept, design, conduct, or reporting of this study. Preparation of ceralasertib for in vitro and in vivo studies is described in the Supplementary Material.

### 4.2. In Vitro X-Irradiation

In vitro X-ray irradiations were performed using an X-RAD 225 generator (Precision X-ray Inc., North Bradford, CT, USA) at 225 kVp, 13.3 mA, at a constant dose rate of 0.59 Gy/min.

### 4.3. Clonogenic Assay, DNA Damage, Flow Cytometry Analysis, and Western Blotting

Clonogenic assay was performed using the protocol of Puck and Markus [49]. Dose-enhancement factors (DEFs) and radiation sensitisation enhancement ratios (RERs) were calculated. RERs of PTEN expression for H460 and A549 models were calculated by dividing the average cell survival of PTEN expressing (NT) + AZD6738 at 8 Gy by the average cell survival of PTEN depleted + AZD6738 at 8 Gy. Differences between NT- and PTEN-depleted groups were compared by using a Student’s *t*-test, and probability values were classified as **** (*p* < 0.0001), *** (*p* < 0.001), ** (*p* < 0.01), and * (*p* < 0.05). A RER less than 1 suggests radio resistance, and an RER greater than 1 indicates an increased radio-sensitisation effect.

DNA damage analysis was performed by quantification of DSBs using immuno-cytochemical staining of 53BP1. Cell cycle distribution was assessed by flow cytometry and protein analysis was performed by Western blotting. Further information on in vitro experiments is provided in the Supplementary Material.

### 4.4. Animals and Maintenance

Tumour response studies were performed by establishing H460 PTEN-depleted cell-line-derived xenografts (CDXs) in 8–10-week-old female SCID mice (Supplementary Material). C3H/HeJ mice at 8–10 weeks old were used to investigate an early inflammatory phenotype indicative of RP. All mice were purchased from Charles River Laboratories (Oxford, UK). All experimental procedures were carried out in accordance with UK home-approved protocols for in vivo experimentation. A minimum of 7 animals per group were used for all in vivo studies.

### 4.5. Pre-Clinical Study Design

All mice received ceralasertib (25 mg/kg delivered by oral gavage once per day, p.o.q.d) prior to RT. Once tumours reached a volume of 100 mm^3^, animals were randomly assigned to one of the experimental arms: vehicle, ceralasertib only, RT only, or ceralasertib + RT. Tumours were irradiated with a total dose of 12 Gy delivered as a single fraction. A parallel opposed beam geometry with a 10 × 10 mm collimator (dose rate of 2.8 ± 0.12 Gy/min) was used to obtain 100% tumour coverage. The modified ellipsoidal formula [50] was used to calculate tumour volumes from caliper measurements across the length, width, and depth of the tumour. When tumour volume reached 600–800 mm^3^, mice were removed (see Supplementary Material for more details).

To evaluate normal lung early inflammatory response, mice were irradiated with a single fraction of 20 Gy using a parallel opposed beam geometry with a 5 × 5 mm collimator (dose rate 0.27 ± 0.16 Gy/min) targeting an isocentre in the upper left lung for minimal displacement during breathing and to spare organs at risk, as previously reported [26].

### 4.6. Histological Assessment of Early Inflammatory Response

To investigate early inflammatory response in the lung as an early indicator of radiation-induced RP, lung samples were obtained at 72 h and 1 and 4 weeks after irradiation. Upon sacrifice, lung tissues were dissected and processed for histology analysis, detailed in the Supplementary Material.

### 4.7. Data Fitting and Statistical Analysis

In vitro radiobiological response data were fitted to Linear Quadratic model of the form SF = exp[−(αD + βD^2^)]. Statistical errors on fit values were calculated as the standard error. Statistical differences were calculated using the Student’s *t*-test. Values are expressed as the mean ± standard deviation. Differences in in vivo tumour growth following different treatments between H460 NT and H460 PTEN-depleted CDX models were assessed using one-way ANOVA. All calculations were performed using GraphPad Prism 7.0 (GraphPad Software, Inc., San Diego, CA, USA). Probability values were classified as **** (*p* < 0.0001), *** (*p* < 0.001), ** (*p* < 0.01), and * (*p* < 0.05).

## 5. Conclusions

In conclusion, this study identified that targeting ATR using ceralasertib enhances the antitumour activity of RT through the induction of DNA damage in PTEN-depleted NSCLC cells, with the addition of ceralasertib having a minimal effect on inflammatory cell infiltration indicative of RP in comparison to radiation exposure alone. Taken together, this study elucidates the potential clinical utility of ceralasertib to enhance the efficacy of RT in NSCLC patients, particularly for those harbouring PTEN-deficient tumours. Gene profiling of patients to identify those with PTEN loss who may benefit from combined ceralasertib-RT treatment may help to improve the prognosis and survival of patients with NSCLC. Our findings provide a pre-clinical and mechanistic rationale for evaluating loss of PTEN in NSCLC as a therapeutic target for ATR inhibition in combination with RT in the clinical setting. It is hoped that the work within our previous publication [26] and the current study will contribute to future clinical studies, including the CONCORDE trial.

The approach for this study utilised shRNA targeting, although other approaches, such as the use of CRISPR knockout PTEN models and investigating the off-target effects of PTEN loss in cancer and resistance to TKIs, could also be applied. To extend the concepts outlined in this paper, further pre-clinical studies should be carried out to investigate the ceralasertib-RT response of NSCLC models with alternative genetic mutations, including ATM and TP53 mutations, which are commonly mutated in NSCLC. Furthermore, applying the findings from this study to different cancer types could increase the clinical application of ceralasertib in combination with RT. Additionally, further investigation into the effect of underlying comorbidities associated with lung cancer and their impact on RP should also be explored for clinical translation.

## Figures and Tables

**Table 1:** Summary of tumour growth delay parameters with uncertainties for H460 NT- and PTEN-depleted isogenic CDXs following treatment with ceralasertib and 12 Gy single fraction radiotherapy exposures, alone and in combination. *p* values were calculated using one-way ANOVA.

Treatment Group	Cell Model		*p* Value (NT- vs. PTEN-Depleted)
	H460 NT Tumour Quadrupling Time (Days)	H460 PTEN-Depleted Tumour Quadrupling Time (Days)	
Vehicle	3 ± 2.53	8 ± 1.6	0.001765
Ceralasertib	19 ± 2.53	10 ± 3.41	0.000406
Vehicle + 12 Gy	25 ± 5.01	13 ± 2.34	0.000340
Ceralasterib + 12 Gy	52 ± 6.07	70 ± 2.24	0.000047

## Data Availability

The original contributions presented in the study are included in the article/Supplementary Material. Further inquiries can be directed to the corresponding author.

## Supplementary Materials

The supporting information can be downloaded at: https://www.mdpi.com/article/10.3390/ijms25147817/s1. Reference [51] is cited in the supplementary materials.
