# Supplementary material for: PTEN Depletion Increases Radiosensitivity in Response to Ataxia Telangiectasia-Related-3 (ATR) Inhibition in Non-Small Cell Lung Cancer (NSCLC)
Source: Int J Mol Sci. 2024 Jul 17;25(14):7817. doi: 10.3390/ijms25147817 (PMC11277409; doi:10.3390/ijms25147817)
Supplement: Supplementary file 1 [file ijms-25-07817-s001.zip › ijms-3049178-supplementary.pdf]

Supplementary Material and Results

Cell culture

H460 cells were routinely cultured in RPMI-1640 medium and A549 cells were cultured in DMEM medium (Lonza). All cell lines were supplemented with 10% fetal bovine serum (FBS) and 1% penicillin-streptomycin (Sigma, UK) and cultured in a humidified atmosphere at 37°C with 5% CO_2. Mycoplasma testing was regularly carried out.

Preparation of ceralasertib

For *in vitro* studies, a 10 mM stock solution of ceralasertib was prepared and stored at -20°C before being diluted in DMSO to the required working concentration. For *in vivo* studies, ceralasertib was dissolved in DMSO at a concentration of 25 mg/mL and diluted 1 in 5 with propylene glycol. An equal amount of water was added to yield 2.5 mg/mL in 10% DMSO, 40% propylene glycol and 50% sterile water. Mice were treated with ceralasertib or drug vehicle at a concentration of 25 mg/kg for 28 days via oral gavage.

Clonogenic Assay Analysis

For clonogenic survival assays, cells were seeded into six-well plates (Sarstedt AG & Co., Germany) at a cell density which was optimal for the specific cell line and absorbed dose. After a 24 h incubation, cells were irradiated with 0-8 Gy of X-rays. For assessment of ceralasertib with irradiation, cells were pre-treated with 100 nM ceralasertib prior to X-rays. For colony formation, cells were incubated for 7-10 days. Colonies were fixed and stained with 2% crystal violet in 80% methanol. Colonies with greater than 50 cells were scored. Plating efficiency percentage (PE) was determined by dividing the number of counted colonies by the number of cells seeded times 100%. Survival fraction was calculated by dividing number of colonies formed post treatment by number of cells seeded, corrected for the PE of cells that were not irradiated.

The DEF was calculated by dividing the required radiation dose to kill 10% of cells by the required radiation dose combined with AZD6738 to kill 10% of cells.

Assessment of DSBs by immuno-cytochemical staining of 53BP1

Cells were seeded on coverslip at a density of 1 x 10^6 and allowed to adhere overnight. Ceralasertib was added to the culture medium at a concentration of 100 nM 1 hour prior to irradiation. After irradiation, cells were incubated for 2, 4, 8 and 24 hours before fixation with a 50% (v/v) mixture of ice cold methanol:acetone solution. Non-irradiated and non-treated cells were used as controls and all cells were stained as previously described [51]. Experiments were performed in at least 3 independent repeats where 50 nuclei were scored per sample.

Cell Cycle profile analysis by flow cytometry

Following treatment with ceralasertib or 2 Gy RT alone or combined, cells were harvested at 48 hours and fixed in 100% ice-cold ethanol and left overnight at 4°C. At the time of analysis, cells were resuspended in 500 µl of PI/RNase A for 30 minutes at 37°C. Flow cytometry was performed on a BD Accuri C6 Plus Flow Cytometer (BD Biosciences, USA). BD Accuri C6 Plus Analysis software was used for quantification.

Western Blotting Analysis

Cell pellets were lysed in 20 mmol/L Tris (pH 8), 200 mmol/L NaCl, 1 mmol/L EDTA, 0.5% (v/v) NP-40, and 10% (v/v) glycerol. Cell lysates were electrophoresed on 10% polyacrylamide gels, transferred onto nitrocellulose membranes and immuno-blotted with phospho-CHK1 (#2348) and anti-PTEN (#9552) (Cell Signalling Technology, USA) at a dilution of 1:1000 in 5% non-fat milk in PBS. Anti-vinculin (#4650) (Sigma, UK) was used as a loading control. The membranes were then washed and developed with Luminata Crescedo Western HRP subtract (Millipore, USA) using the GBox Imager by Syngene (Cambridge, UK).

Preclinical study design

All mice were irradiated with 225 kVp X-rays under cone beam computed tomography (CBCT) image guidance using a small animal radiotherapy research platform (SARRP, Xstrahl Inc., Suwanee, GA). Studies were designed to assess tumour control and radiation induced toxicity using multiple radiotherapy schedules in combination with ceralasertib or drug vehicle. Ceralasertib was delivered at a concentration of 25 mg/kg for 28 days or drug vehicle.

Establishment of cell line derived xenografts (CDXs) and calculation of tumour volume

H460 CDXs were established by sub-cutaneous injection of 5 x 10^6 cells suspended in 100 µl of a 50% (v/v) mixture of culture media and Matrigel. Mice were enrolled to the study once a tumour volume of 100 mm^3 had been reached. All animals were housed 5 per cage under pathogen-free conditions and allowed to acclimatise for a minimum period of 1 week prior to implantation. Animals received a standard laboratory diet and water *ad libitum*.

Tumour volumes were measures using electronic callipers to record 3 orthogonal measurements across the tumour (length, breadth, height) and the volume calculated from the gross mean diameter (GMD) according to the form *tumour volume* = $\frac{4}{3}\pi r^3$. Data is presented as median tumour volume for the experimental group ± standard error.

Histological assessment of macrophages and neutrophils

Slides were stained with F4/80 Macrophage (ab6640) and NIMP-R14 Neutrophils (ab2557) antibodies (Abcam, UK). All slides were warmed to 60˚C to melt paraffin and subjected to a series of deparaffinization and rehydration steps. Antigen retrieval was performed in sodium citrate buffer pH 6.0 and peroxidase activity was blocked in 3% hydrogen peroxide in methanol for 15 minutes. All slides were then incubated in blocking buffer made in 10% FBS (Sigma, UK) for one hour. Primary antibodies were used at a dilution of 1:200 (ab6640) and 1:100 (ab2557) in blocking buffer incubating overnight at 4˚C. Samples were then washed in phosphate buffered saline (PBS, Sigma, UK) solution containing 0.05% Tween20 (Sigma, UK). Antibody signal was detected using Anti-Rat IgG, Mouse Adsorbed Reagent (Vector Labs (MP-7444), visualized with DAB substrate kit (ab64238) and counterstained with haematoxylin (Sigma, UK). Inflammation was defined by the presence of neutrophils and macrophages and sections were scored for the number of inflammatory cells present in a 0.25 mm^2 area at a 10x magnification.

Data fitting and statistical analysis

In vitro radiobiological response data was fitted to Linear Quadratic model of the form SF = $\exp[-(\alpha D+ \beta D^2)]$. Statistical errors on fit values were calculated as the standard error. Statistical differences were calculated using the Student's t-test. Values are expressed as the mean ± standard deviation. Differences in *in vivo* tumour growth following different treatments between H460 NT and H460 PTEN-depleted CDX models was assessed

using one-way ANOVA. All calculations were performed using GraphPad Prism 7.0 (GraphPad Software, Inc.). Probability values were classified as **** (p < 0. 0001), *** (p < 0. 001), ** (p < 0.01) and * (p < 0.05).

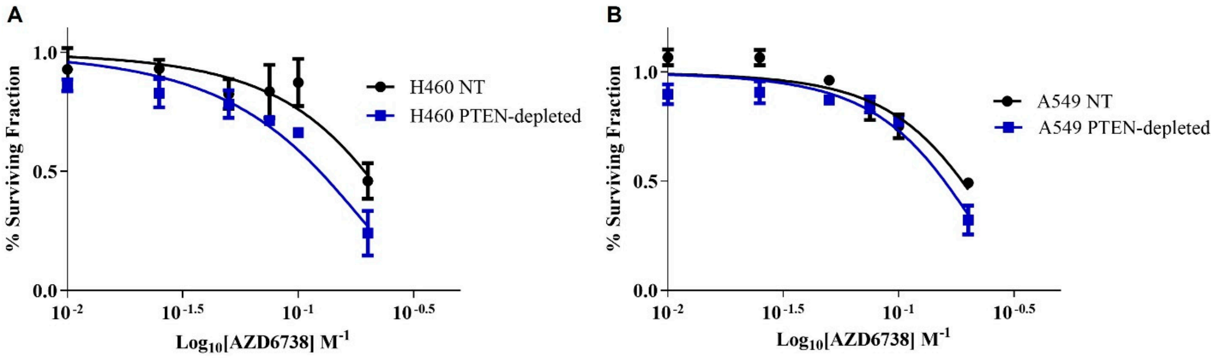

Figure S1. Cellular toxicity of ceralasertib in (A) H460 and (B) A549 human prostate cancer cell lines was determined using clonogenic survival curves. Cells were treated with a dose range of 0.01-0.2 μM. Surviving fraction was normalised to the untreated control. Experiments were performed in triplicate on three independent occasions.

Table S1. IC50 values of clonogenic survival assays for H460 and A549 NT and PTEN-depleted models indicating the ceralasertib concentration at which cell survival was reduced by 50%.

	**H460 NT**	**H460 PTEN-depleted**	**A549**	**A549 PTEN-depleted**
**IC_{50} Value**	0.19 μM	0.12 μM	0.19 μM	0.16 μM

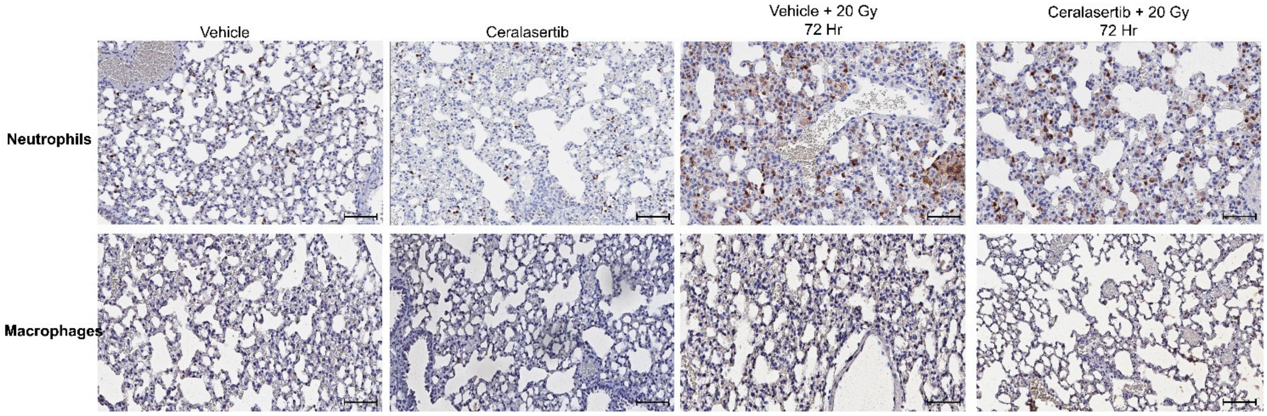

Figure S2. Representative images of immunohistochemistry analysis of F4/80 Macrophage and NIMP-R14 Neutrophils in lung tissue sections from C3H/NeJ mice at 72 hours after irradiation. Scale bar represents 100 μM length.

Table S2. Radiobiological parameters (± SEM) of H460 and A549 NT and PTEN-depleted cells with and without ceralasertib after fitting to a Linear Quadratic model. SF2 and SF4 represent survival fractions at doses 2 Gy and 4 Gy. Differences between RT and RT + Ceralasertib were calculated by using a Student's *t*-test. Also presented are DEFs.

	**H460 Cell Line**				**A549 Cell Line**			
	NT	NT+ Ceralasertib	PTEN-depleted	PTEN-depleted +Ceralasertib	NT	NT + Ceralasertib	PTEN-depleted	PTEN-depleted + Ceralasertib
α	0.11 ± 0.02	0.35 ± 0.10	0.15 ± 0.03	0.23 ± 0.13	0.19 ± 0.10	0.36 ± 0.12	0.19 ± 0.06	0.62 ± 0.42
β	0.06 ± 0.002	0.04 ± 0.01	0.05 ± 0.004	0.07 ± 0.01	0.03 ± 0.01	0.02 ± 0.01	0.04 ± 0.008	0.02 ± 0.15
DEF	1.5		1.32		1.03		1.73	
SF2	0.67 ± 0.07	0.28 ± 0.03	0.69 ± 0.03	0.40 ± 0.2	0.5 ± 0.07	0.46 ± 0.05	0.61 ± 0.1	0.26 ± 0.1
	$p = 0.0009$		$p = 0.0679$		$p = 0.4657$		$p = 0.0128$	
SF4	0.45 ± 0.05	0.17 ± 0.03	0.38 ± 0.007	0.11 ± 0.02	0.26 ± 0.02	0.23 ± 0.04	0.39 ± 0.09	0.05 ± 0.01
	$p = 0.0011$		$p < 0.0001$		$p = 0.3099$		$p = 0.0029$	

Table S3. Radiation Sensitisation Enhancement Ratio of PTEN expression in combination with Ceralasertib.

	H460	A549
**RER**	2.17 ± 0.04 ($p < 0.01$)	4.05 ± 0.09 ($p < 0.01$)

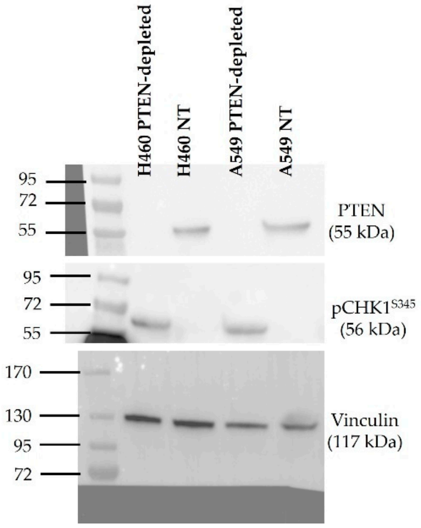

Figure S3. Whole western blots including molecular weight markers for protein expression of phospho-Chk1 and total PTEN in H460 and A549 NSCLC PTEN isogenic cell models. Vinculin was used as a loading control.
